# A meta-analysis of copper level and risk of preeclampsia: evidence from 12 publications

**DOI:** 10.1042/BSR20160197

**Published:** 2016-08-24

**Authors:** Yuqin Fan, Yan Kang, Min Zhang

**Affiliations:** * Maternal and Child Health Institute of Shandong Province, Jinan 250021, China; † Department of Obstetrics and Gynecology, Shandong Provincial Hospital Affilated to Shandong University, Jinan, 250021, China

**Keywords:** copper level, meta-analysis, preeclampsia

## Abstract

The association between copper level and risk of preeclampsia (PE) has produced inconsistent results. Thus, a meta-analysis was conducted to summarize the evidence from epidemiological studies for copper level and PE risk. Pertinent studies were identified by a search of PubMed and Web of Knowledge up to April 2016. Standardized mean difference (SMD) was performed to combine the results. Random-effect model (REM) was used. Publication bias was estimated using Egger's regression asymmetry test. Twelve articles (10 case–control studies and 2 cross-sectional studies) involving 442 PE cases and 463 health controls were included in this meta-analysis. Our pooled results suggested that PE patients had a higher copper level compared with healthy pregnancy controls [summary SMD=0.69, 95% CI: 0.54–0.84, *I*^2^=96.7%; *P*<0.001]. The association was also significant in Asian population [SMD=0.73, 95% CI=0.57–0.90, *I*^2^=97.3%] and European populations [SMD=0.50, 95% CI=0.14–0.86, *I*^2^=58.9%]. After conducting the subgroup analysis and sensitive analysis, the results showed consistent significant association with the one based on all studies. No publication biases were found. Our analysis indicated that plasma or serum copper level in PE patients was significantly higher than that in healthy pregnancy women.

## INTRODUCTION

Preeclampsia (PE) is defined as a progressive, multisystemic disorder which characterized by triad of high-blood pressure to the extent of 140/90 mm Hg or more, oedema, as well as proteinuria, developing after 20 weeks of gestation [1]. And PE is one of the most common medical complications during pregnancy. The incidence of PE in pregnancies fluctuates ranging from 2% to 8% in the word [2,3]. Furthermore, PE is also the leading cause of both perinatal and maternal morbidity and mortality in the world [4]. Complications of PE are the third leading cause of pregnancy-related deaths, just follows after haemorrhage and embolism among pregnancy-related cause of death [5]. PE is associated with increased risks of placental abruption, cerebrovascular and cardiovascular complications, acute renal failure, disseminated intravascular coagulation and maternal death [5]. Although it has been extensive studied, its specific pathophysiology and aetiology remain obscure [6,7].

Copper is an important trace element which takes parts in structure of many enzymes like lysyl oxidase, cytochrome coxidase, tyrosinase, dopamine-β-hydroxylase, peptidylgly-cine alpha-amidating monooxygenase, monoamine oxidase, ceruloplasmin and copper–zinc superoxide dismutase (SOD) [8]. Many different studies have shown that there is an association between occurrence of PE and trace elements, however, the results are not consistent. To our knowledge, no meta-analysis has been conducted on the question. Given these reasons, we performed this meta-analysis to: (1) first assess the relationship between blood copper level and PE; (2) explore the potential heterogeneity among studies and (3) explore the potential publication bias.

## MATERIALS AND METHODS

### Literature search and selection

We performed a systematic literature search from January 1990 to April 2016 using the databases of PubMed and Web of Science, using the following search terms: ‘copper’ and ‘preeclampsia’ to search the English related articles without restrictions. Moreover, we also reviewed the references of the included studies and review articles to identify additional studies which were not captured by our database searches.

### Inclusion criteria

The inclusion criteria were as follows: (1) observational studies design; (2) the exposure of interest was copper level; (3) the outcome of interest was PE; (4) the blood sample was venous blood taken from cases and controls after the 20th week of pregnancy; (5) data of the plasma or serum copper levels are available in the results or can be obtained from the corresponding author; (6) data were presented as mean ± standard deviation (S.D.) (or data provided from which mean and S.D. could be calculated). Accordingly, we excluded reviews, letters, editorials and RCT studies.

All identified studies were carefully reviewed independently by two investigators to identify and determine whether an individual study was eligible for inclusion criteria in this meta-analysis. If the two reviewers could not reach a consensus about the eligibility of an article, it was resolved by disputing with a third investigator.

### Data extraction

Two investigators independently performed the search, reviewing all identified studies and collecting data. The following data were extracted: the mean ± S.D. on plasma or serum copper levels, first authors’ name, publication year, country where the study was performed, follow-up duration, mean age, criteria for PE diagnosis, sample size, fasting status before blood collection, determination method of plasma or serum copper levels and other information. If given the standard error mean (S.E.M.) of copper level in the study, the S.D. is calculated by the following formula: S.E.M.=S.D./
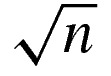
. Because the underlying units of measurement varied from study to study, all units were converted to ug/l.

### Statistical analysis

Pooled measure was calculated to assess the strength of association between plasma or serum copper level and PE. Because of the underlying units of measurement and experiment conditions varied from study to study, all statistical analyses were performed using standard mean difference (SMD) with 95% CI to assess the strength of association between copper level and PE [9]. The *I*^2^ was used to assess heterogeneity (*I*^2^ values of 0, 25, 50 and 75% represent no, low, moderate and high heterogeneity respectively) [10]. The random-effect model (REM) was adopted to calculate the combined results [11]. Meta-regression was performed to assess the potentially important covariates that might exert substantial impacts on between-study heterogeneity. The subgroup analyses (study type, geographic locations, sample type and fasting status) were also conducted to explore the between-study heterogeneity. A sensitivity analysis was performed with one study removed at a time to assess whether the results could have been affected markedly by a single study. Publication bias was investigated with visual inspection of the Egger test. All statistical analyses were performed with Stata 10.0 (Stata Corporation, College Station, TX, USA). All reported probabilities (*P* values) were two-sided with *P*<0.05 considered statistically significant. The significance level for the tests of heterogeneity and publication bias is set at 0.1.

## RESULTS

### Characteristics of studies

**Figure 1:**
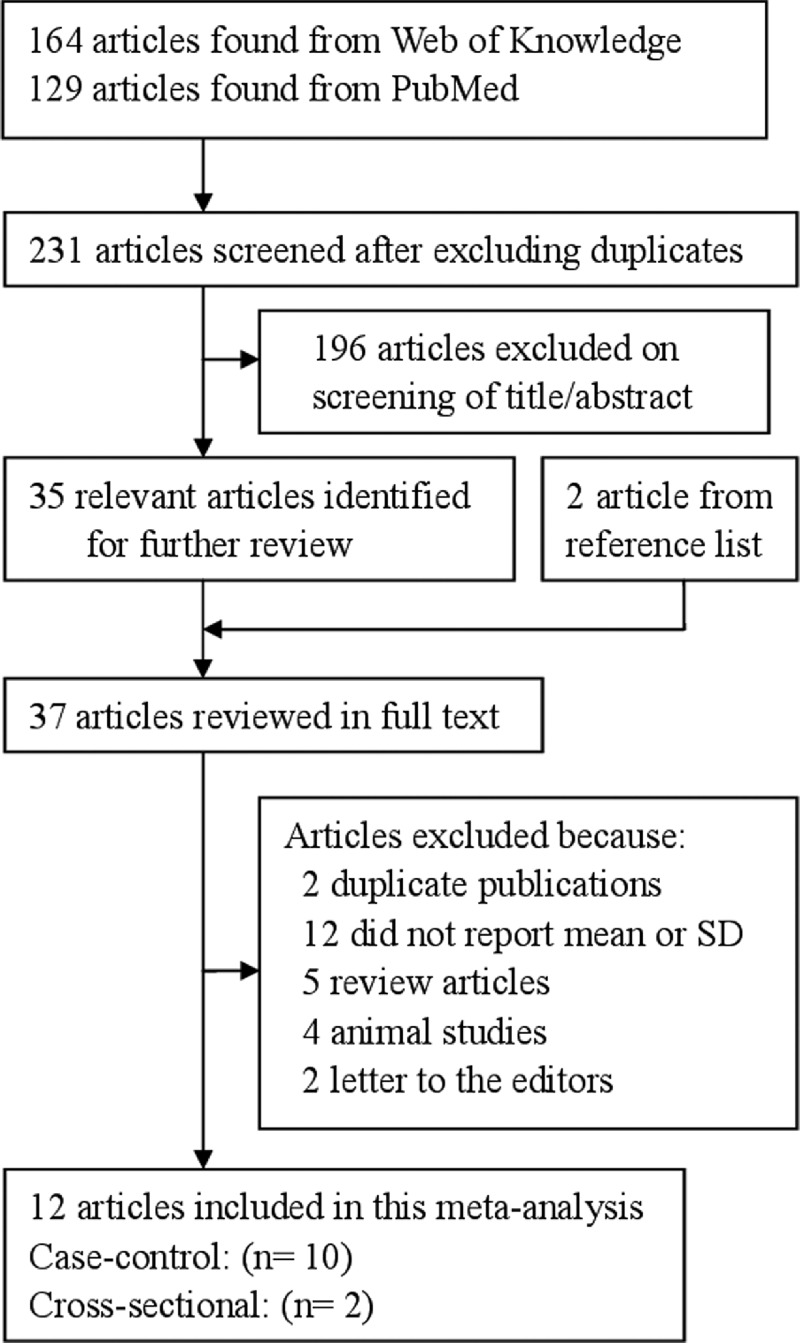
The flow diagram of screened, excluded and analysed publications

The detailed steps of our literature search are shown in Figure 1. After the application of search strategy, we found that a total of 12 relevant articles [12–23] appeared to fulfil the inclusion criteria. All of the full texts, published between 1990 and 2016. All included studies are observational study, including case–control study and cross-sectional study. The pooled subjects included a total of 463 healthy pregnancy controls and 442 PE cases. The baseline characteristics of the study participants and design characteristics in the published articles are shown in Table 1.

**Table 1:** Characteristics of all included studies

				Preeclampsia		Health control			
Study, year	Country	Age (mean ± S.D.)	Study type	*n*	Cu: mean ± S.D. (μg/l)	*n*	Cu: mean ± S.D. (μg/l)	Sample type	Fasting status
Açikgoz et al., 2006	Turkey	24.90±2.50	Case–control	16	2697±867	20	2810±884	Serum	No
Ahsan et al., 2013	Bangladesh	26.05±5.41	Cross-sectional	44	1105±130	27	1040±130	Serum	No
Atamer et al., 2005	Turkey	27.00±3.89	Cross-sectional	32	2612±472	28	1458±396	Serum	Overnight fast
Bakacak et al., 2015	Turkey	29.20±3.56	Case–control	38	1995±263.58	40	1524.5±133.64	Serum	Overnight fast
Borella et al., 1990	Italy	34-37	Case–control	24	3365.7±1245.4	35	2622.1±575.25	Plasma	Yes
Farzin et al., 2012	Iran	27.43±3.91	Case–control	60	1182.8±169.2	60	1165.5±152.3	Serum	Yes
Fenzl et al., 2013	Croatia	31.20	Case–control	30	2204.15±532.35	37	2082.6±476.45	Serum	Overnight fast
Ilhan et al., 2002	Turkey	25.00	Case–control	21	1767.3±259.1	30	1581.5±278.6	Plasma	Overnight fast
Kolusari et al., 2008	Turkey	27.91±5.21	Case–control	47	26.5±7.6	48	20.8±4.6	Serum	Overnight fast
Rafeeinia et al., 2014	Iran	26.50±3.90	Case–control	50	2400±640	50	1300±340	Serum	Overnight fast
Sarwar et al., 2013	Bangladesh	25.46±0.85	Case–control	50	1980±100	58	2580±60	Serum	8 h fasting condition
Serdar et al., 2006	Turkey	25.00±2.30	Case–control	30	1880±480	30	1590±380	Serum	No

### Plasma or serum copper level and PE

**Figure 2:**
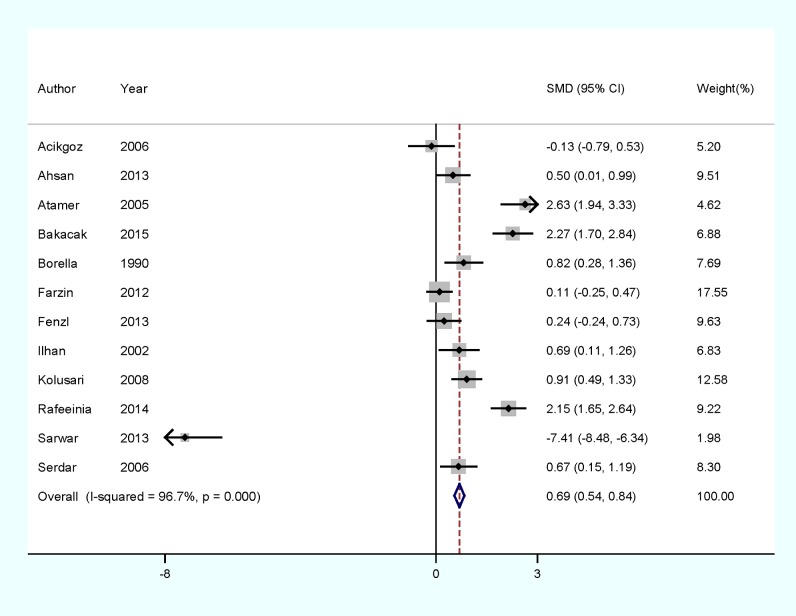
The forest plot of the association between copper level and PE risk

Eight of 12 included studies reported that copper levels were significantly higher in PE patients than controls, whereas 3 studies found no significant difference. However, Sarwar et al. [22] reported that copper levels were significantly lower in PE patients than controls. Of all included studies, the combined analysis results of 12 studies which showed all PE cases compared with healthy pregnancy between the copper level and PE was shown in forest plots (Figure 2). When compared with healthy pregnancy controls, PE patients had a higher copper level. The pooled SMD from a REM was 0.69 ug/l (95% CI: 0.54–0.84, *I*^2^=96.7%; *P*< 0.001).

### Meta-regression and subgroup analyses

As seen in Figure 2, strong evidence of heterogeneity among studies was demonstrated for plasma or serum copper and PE. However *P* values of univariate meta-regression analysis with the covariates of publication year, study type, fasting status of participants and geographic locations were 0.612, 0.241, 0.572 and 0.386 respectively. The results showed no covariates having a significant impact on between-study heterogeneity.

Table 2 showed the results from subgroup analyses. Within all subgroups, the pooled SMD was consistently significant; indicating that copper level was higher in patients with PE than healthy pregnancy controls. The associations were significant both in the case-control studies [SMD=0.61, 95% CI=0.45–0.77, *I*^2^=97.0%] and cross-sectional studies [SMD=1.20, 95% CI=0.80–1.60, *I*^2^=95.9%]. The pooled SMD were significant in studies conducted among Asian populations [SMD=0.73, 95% CI=0.57–0.90, *I*^2^=97.3%] and European populations [SMD=0.50, 95% CI=0.14–0.86, *I*^2^=58.9%]. We also conducted a subgroup analysis stratified by fasting status of participants, the pooled SMD was 0.77 (95% CI=0.60–0.95, *I*^2^=97.6%) in the subgroup of fasting. The pooled SMD were 0.68 (95% CI=0.52–0.84, *I*^2^=97.3%) in the serum-based sample subgroup and 0.76 (95% CI=0.36–1.15, *I*^2^=0.0%) in the plasma-based sample subgroup.

**Table 2:** Summary SMD for overall and subgroup results

Subgroup	Cases	Studies	SMD (95% CI)	*I*^2^ (%)	*P*_heterogeneity_
All studies	442	12	0.69 (0.54–0.84)	96.7	0.000
Study type					
Case–control	366	10	0.61 (0.45–0.77)	97.0	0.000
Cross-sectional	76	2	1.20 (0.80–1.60)	95.9	0.000
Geographic locations					
Asia	388	10	0.73 (0.57–0.90)	97.3	0.000
Europe	54	2	0.50 (0.14–0.86)	58.9	0.000
Sample type					
Plasma	45	2	0.76 (0.36–1.15)	0.0	0.740
Serum	397	10	0.68 (0.52–0.84)	97.3	0.000
Fasting status					
Yes	352	9	0.77 (0.60–0.95)	97.6	0.000
No	90	3	0.42 (0.11–0.73)	45.4	0.160

### Sensitivity analysis and publication bias

As sensitivity analyses, we excluded one study at a time to assess the stability of the results. There was no significant change in the pooled SMD or 95% CI on excluding any of the studies. This means no individual study had excessive influence on the pooled effect between risk of plasma or serum copper and PE risk. The Egger test showed no evidence of significant publication bias for the analysis between plasma or serum copper level and PE for all included studies. The *P* value of Egger test was 0.268.

## DISCUSSION

In recent years, many studies had been performed to evaluate the relationship between blood copper level and PE based on populations. To our knowledge, our study is the first meta-analysis to discuss the association between plasma or serum copper level and PE. Our meta-analysis contained 12 studies, including 463 healthy pregnancy controls and 442 PE cases, identified that the plasma or serum copper level in PE patients was significantly higher than that in healthy pregnancy women.

PE is a multisystem and multifactorial disease that affects both mother and foetus by vascular dysfunction and by intrauterine growth restriction [24]. Although there has been implicated many pathophysiological factors in the aetiology of PE, its aetiology is still under investigation [25]. It is reported that excessive lipid peroxidation or oxidative stress is an important determinant of PE [24]. Oxidative stress contributes to maternal endothelial cell activation and enhanced apoptosis of trophoblasts and is believed to underlie the intense vasoconstriction and procoagulant state of PE [25–27]. Copper as an antioxidant trace metals, can alleviate oxidative stress by scavenging free radicals or function as essential substrates or cofactors for the adequate activation of antioxidant enzymes, such as SOD. In addition, Cu deficiency has a negative effect on Cu–Zn SOD enzyme system [28]. Cu–Zn SOD acts as antioxidant defence system. Subsequently impaired Cu–Zn SOD activity contributes to the oxidative damage in the body which may worsen several disease states [29].

Between-study heterogeneity is common in meta-analysis [30], and it is essential to explore the potential sources of between-study heterogeneity. An indeterminate number of characteristics that varied among the studies could be the sources, such as publication year, study type, fasting status of participants, geographic locations and other variation of the covariates in studies. Thus we used both meta-regression and sensitive analysis that aimed to explore the potential important causes of the between-study heterogeneity. Our meta-analysis did not find the covariates of publication year, study type, fasting status of participants and geographic locations as the important contributors to the between-study heterogeneity. When we conducted the subgroup analysis, the between-study heterogeneity was also persisted in some subgroups. The potential contributors to the conflicting results could be that: (1) the included studies with different blood sample handling methods and preservation methods; (2) the potential confounders adjusted in each study were diverse etc.

As a meta-analysis of relevant published studies, our study has several strengths. First, the large numbers of participants allow a much greater possibility of reaching reasonable conclusions and conducting subgroup analysis. Second, the literatures search and data extraction was conducted independently by two investigators; therefore, the random error introduced by inserting data into tables was avoided greatly. Third, we set a strict inclusion criterion to control the quality of including studies. Fourth, after conducting the subgroup analysis and sensitive analysis, the results showed consistent significant association with the one based on all studies, strongly identifying the association stable.

However, our study also has limitations. First, significant heterogeneity existed overall. To clarify the sources of heterogeneity, we conducted the subgroup and sensitivity analyses. However, residual confounding factors across studies remain as a cause for concern in this meta-analysis. Therefore, heterogeneity was still an inevitable problem that may affect the precision of overall results. Second, we found significant associations between copper levels with the risk of PE both in the Asian populations and European populations. However, only two studies came from Europe, so, more studies originating in Europe and other countries are required to investigate the association between copper levels and PE risk. Due to this limitation, the results are applicable to the Asia, but cannot be extended to populations elsewhere. Finally, although we only included the article written in English and did not search grey literature, the Egger test showed no evidence of significant publication bias for the analysis between plasma or serum copper level and PE for all included studies. The *P* value of Egger test was 0.268.

In summary, results from this meta-analysis indicate that plasma or serum copper level in PE patients was significantly higher than that in healthy pregnancy women.

## Abbreviations

PE: preeclampsia
REM: random-effect model
SMD: standardized mean difference
SOD: superoxide dismutase
